# Perception and Reality: Why a Wholly Empirical Paradigm is Needed to Understand Vision

**DOI:** 10.3389/fnsys.2015.00156

**Published:** 2015-11-18

**Authors:** Dale Purves, Yaniv Morgenstern, William T. Wojtach

**Affiliations:** ^1^Duke Institute for Brain Sciences, Duke UniversityDurham, NC, USA; ^2^Duke-NUS Graduate Medical SchoolSingapore, Singapore

**Keywords:** vision, visual perception, feature detection, Bayesian probability, efficient coding, empirical ranking

## Abstract

A central puzzle in vision science is how perceptions that are routinely at odds with physical measurements of real world properties can arise from neural responses that nonetheless lead to effective behaviors. Here we argue that the solution depends on: (1) rejecting the assumption that the goal of vision is to recover, however imperfectly, properties of the world; and (2) replacing it with a paradigm in which perceptions reflect biological utility based on past experience rather than objective features of the environment. Present evidence is consistent with the conclusion that conceiving vision in wholly empirical terms provides a plausible way to understand what we see and why.

## Introduction

A widely accepted concept of vision in recent decades stems from studies carried out by Stephen Kuffler, David Hubel and Torsten Wiesel beginning in the 1950s (Kuffler, 1953; Hubel and Wiesel, 2005). This seminal work showed that neurons in the primary visual pathway of cats and monkeys respond to light stimuli in specific ways, implying that the detection of retinal image features plays a central role in visual perception. Based on the properties of simpler input-level cells, Hubel and Wiesel discovered that neurons in V1 respond selectively to retinal activation elicited by oriented bars of light, bars of a certain length, bars moving in different directions, and stimuli with different spectral properties. These and other findings earned Hubel and Wiesel a Nobel Prize in 1981 (Kuffler had died in 1980), and inspired a generation of scientists to pursue similar electrophysiological and neuroanatomical research in a variety of species in the ongoing effort to reveal how vision works.

A seemingly straightforward interpretation of these observations is that the visual system operates analytically, extracting features from retinal images, efficiently filtering and processing image features in a series of computational steps, and ultimately combining them to provide a close approximation of physical reality that is then used to guide behavior. This concept of visual perception is logical, accords with electrophysiological and anatomical evidence, and has the further merit of being similar to the operation of computers, providing an analogy that connects biological vision with machine vision and artificial intelligence (Marr, 1982). Finally, this interpretation concurs with the impression that we see the world more or less as it really is and behave accordingly. Indeed, to do otherwise would seem to defy common sense and insure failure.

**Figure 1:**
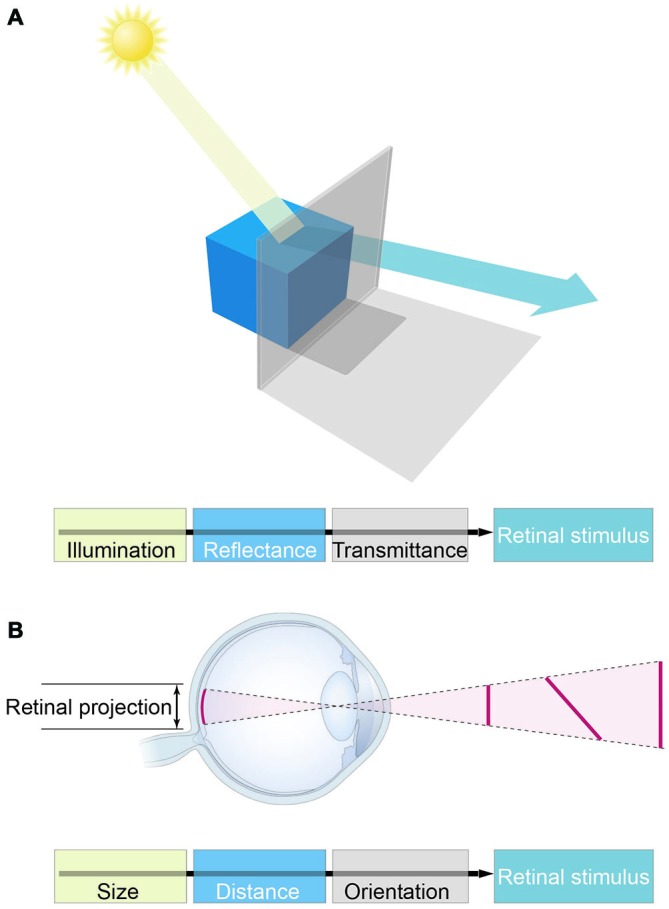
**The major obstacle to the concept of vision as feature representation. (A)** Luminance values in retinal stimuli are determined by illumination and reflectance, as well as a host of other factors (e.g., atmospheric transmittance, spectral content, and many more). These physical parameters are conflated in light stimuli, however, precluding biological measurements of the objective world in which perceptions and behaviors must play out. **(B)** The analogous conflation of geometrical information in retinal stimuli.

**Figure 2:**
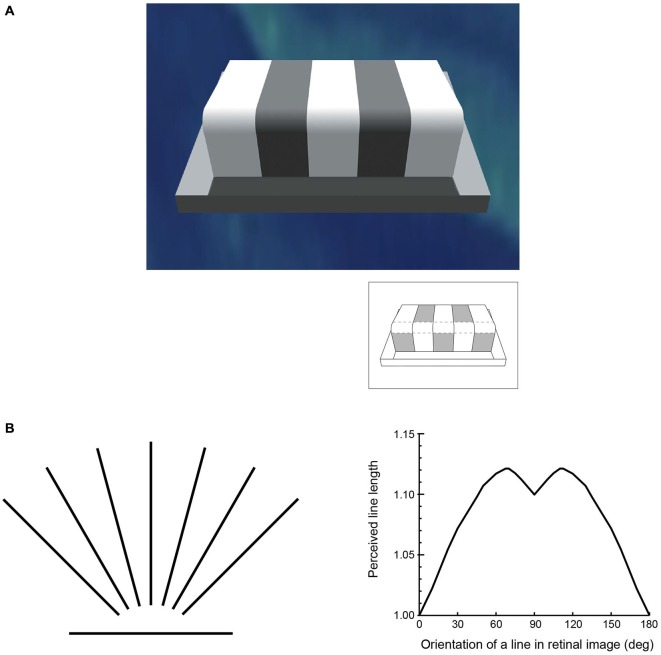
**The perception of basic visual qualities is at odds with the world assessed by physical instruments. (A)** One of many examples generated over the last century or more illustrating the discrepancy between luminance and lightness. Although each of the patches indicated in the inset returns the same amount of light to the eye (i.e., they have the same luminance), their apparent lightness values in the scene are very different. **(B)** An example of the discrepancy between perceived and measured geometry that has again been repeatedly documented since the mid-19th century. The lines on the left are all of equal length, but, as shown on the right, are perceived differently depending on their orientation (apparent length is expressed in relation to the horizontal line, which is seen as shortest in psychophysical testing).

Attractive though it is, this interpretation fails to consider an axiomatic fact about biological vision: retinal images conflate the physical properties of objects, and therefore cannot be used to recover the objective properties of the world (Figure 1). Consequently, the basic visual qualities we perceive—e.g., lightness, color, form, distance, depth and motion—cannot specify reality. A further fact that adds to the challenge of understanding how vision works is the discrepancy between these perceived qualities and the physical parameters of objects and conditions in the world (Figure 2). As numerous psychophysical studies have shown, lightness and darkness percepts are at odds with luminance, color is at odds with distributions of spectral power, size, distance and depth are at odds with geometrical measurements, and speeds and directions of motion are at odds with measured vectors (Gelb, 1929; Stevens, 1975; Rock, 1984; Robinson, 1998; Purves and Lotto, 2003; Wojtach et al., 2008, 2009; Sung et al., 2009; Purves et al., 2014). These differences between perception and reality cannot be dismissed as minor errors or approximations that are “close enough” to succeed, since the discrepancies are ubiquitous and often profound (see Figure 2A, for example).

The result has been diminished confidence in concepts of vision based on retinal feature detection, opening the door to other ways of understanding visual perception, the purposes of visual circuitry, and the genesis of visually guided behavior. A common denominator of these alternative views is the use of past experience—i.e., empirical evidence—to explain vision.

## Early Ideas About Vision on an Empirical Basis

The loss of information due to the transformation of three-dimensional (3-D) Euclidean space into two-dimensional (2-D) images and the introduction of noise inherent in biological processes led some early schools of psychology to advocate theories of vision that included the influence of lifetime experience. This line of thinking began in the mid-19th century when Hermann von Helmholtz proposed that perceptions arising from impoverished images are supplemented by “unconscious inferences” about reality made on the basis of individual experience (Helmholtz, 1866/1924). He added the qualifier “unconscious” because observers are rarely aware of how their past experience could affect perception.

For much of the first half of the 20th century the role of empirical information in determining perception was conceived in terms of gestalt laws or other heuristics. The gestalt school was founded shortly after the turn of the century by Max Wertheimer (1912/1950), and advanced under the aegis of his students Kurt Koffka (1935) and Wolfgang Köhler (1947). At the core of gestalt theory is the idea that the “units of experience go with the functional units in the underlying physiological processes” (Wolfgang Köhler, 1947, p. 63). In gestalt terms, this influence was codified as the “the law of präganz” (meaning “conciseness”), expressing the idea that, based on experience, any perception would be determined by the simplest possible source of the image in question. Building on some of these ideas Egon Brünswik argued further that, in order to fully understand perception, the connection between the organism and the environment must be clarified. Given that information acquired by sense organs is uncertain, he supposed that visual animals must rely on the statistical nature of environments to achieve their goals. As described in his theory of “probabilistic functionalism” (Brünswik, 1956/1997), Brünswik anticipated some current empirical approaches to vision based on probable world states (see Vision as Bayesian Inference).

Brünswik’s emphasis on the environment influenced the subsequent work of James Gibson (1966, 1979), who carried empirical thinking in yet another direction by arguing that perception is determined by the objects and circumstances observers are exposed to when moving though the world. Gibson proposed that observers could directly perceive their environment by relying on “invariances” in the structure of retinal images, a position similar to the statistical regularity of objects and conditions (e.g., commonly encountered ratios, proportions, and the like) identified by Brünswik. The invariances used by agents exploring the world led Gibson to posit vision as a “perceptual system” that included both the body and its environment—a position fundamentally different from Helmholtz’s idea of empirically modifying retinal image information acquired by a “sensing system”. In the case of size and distance, for example, Gibson maintained that the ratio of object projections to background textures provided the kind of invariant information that would allow an observer to directly apprehend otherwise ambiguous size-distance relationships. He took the mechanism to be one of “resonance” between the activity of a perceptual system and the properties of the environment that gave rise to light stimuli.

Although these early empirical strategies were imaginative and in some ways prescient, they suffered from an absence of ties to the structure and function of animal visual systems. Thus Helmholtz, Wertheimer, Koffka, Köhler, Brünswik, and Gibson were necessarily vague, speculative or simply mute about how empirical information might be usefully implemented in visual system physiology and anatomy. In consequence, empirical approaches to vision began to languish at mid-century, while visual neurobiology with its increasingly concrete evidence about how visual systems operate at the neuronal level came to dominate vision science in the 1960s and for the next several decades (Hubel and Wiesel, 2005).

By the 1990s, however, it was becoming increasingly apparent that, despite key insights into the feature-selective properties of visual neurons, neurophysiological and neuroanatomical approaches to perception were unable to explain how processing retinal image features could, in principle, contend with the inability of visual stimuli to convey information about the objective properties of the world (see Figure 1). At the same time, advances in computer hardware and software were rapidly making the evaluation of large datasets relatively easy. Accordingly, investigators began to re-examine the merits of vision determined by past experience. The basis of much of this thinking has been that visual perception could be understood as probabilistic inferences about the most likely physical states of the world.

## Vision as Bayesian Inference

The most popular approach to vision as statistical inference is based on Bayesian decision theory (Knill and Richards, 1996; Mamassian et al., 2002; Kersten and Yuille, 2003; Lee and Mumford, 2003; Kersten et al., 2004; Knill and Pouget, 2004; Körding, 2014). In effect, investigators built on Helmholtz’s idea of unconscious inference, formally recasting it in terms of Bayes’ theorem (Bayes, 1763), a widely used procedure for assessing the probability of an inference being correct given a set of inconclusive evidence. The theorem states that the probability of inference *A* being true given evidence *B* (the posterior probability) depends on the probability of obtaining *B* given inference *A* (the likelihood), multiplied by the probability of inference *A* being true (the prior), these factors typically being normalized by dividing by the probability of evidence *B*. Thus the theorem can be written as:

(1) $$p(A|B)=\frac{p(B|A)p(A)}{p(B)}$$

To illustrate a Bayesian approach to vision, consider a simple example in which an image is generated by a single light source and a given surface reflectance (e.g., Brainard, 2009; see also Allred and Brainard, 2013). Although many physical factors are involved in generating natural images (see Figure 1A), the luminance values (L) in an image are primarily the product of the intensity of illumination (I) and reflectance properties of surfaces (R). Thus the first step in validating the idea that vision follows Bayes’ theorem would be to determine the probability distributions of surface reflectance and illumination values—the priors *p*(R) and *p*(I), respectively—which can be approximated by measurements in the environment. The next step would be to derive the likelihood function *p*(L|R, I), i.e., the probability of a specific luminance being generated by various surface reflectance and illumination intensities. The posterior distribution, *p*(R, I|L), is then obtained by multiplying the prior distribution by the likelihood function:

(2) $$p(R,I|L)=\frac{p(L|R,I)p(R)p(I)}{p(L)}$$

Because the posterior distribution indicates only the relative probabilities of a set of possible sources, a final step is to select particular reflectance and illumination values from the set according to an assumed gain-loss function. The perceptual outcome—the lightness seen—would presumably accord with the surface reflectance at the most likely combination of surface reflectance and illuminant intensity values. Thus, perceived lightness is taken to be an estimate of surface reflectance.

Experimental assessments of the responses to visual stimuli are made in terms of a Bayesian “ideal observer”, defined as an observer who always responds to the most probable state of the world (Geisler, 2011)—e.g., the most probable surface reflectance value that could have given rise to a retinal luminance value. As indicated in equation (2), an experimenter can validate how well humans approach this ideal by measuring perceptual estimates—in this case, by gauging perceived lightness, which is assumed to be an estimate of surface reflectance—and comparing these to predictions that combine stimulus information in a statistically optical fashion (e.g., Ernst and Banks, 2002; Weiss et al., 2002). Studies of this sort have supported the conclusion that vision can indeed be modeled as a system based on Bayesian inferences. Whether estimating surface slant (Knill and Saunders, 2003), responding to apparent motion stimuli (Weiss et al., 2002; Stocker and Simoncelli, 2006), planning movements (Körding and Wolpert, 2004; Tassinari et al., 2006), integrating somatosensory haptics and visual cues (Ernst and Banks, 2002), combining prior real world assumptions with those in the scene at hand (Morgenstern et al., 2011), or reporting lightness (Allred and Brainard, 2013), subjects perform at close to Bayesian optimality.

The compelling logic of Bayesian decision theory and its useful formalization of Helmholtz’s concept of empirical inference notwithstanding, Bayesian approaches that rely on estimating properties of the world are at a loss when seeking to understand visual neurobiology and/or the neural mechanisms underlying psychophysical functions. The reason is simply that biological visual systems cannot acquire the information that Bayesian decision theory demands: when a Bayesian ideal observer predicts perception, it is because the perceived quality is assumed to estimate the actual properties and conditions in the world. Given the inherent ambiguity of retinal images (see Figure 1), however, Bayesian priors and likelihoods of reflectance, illumination or other physical variables are not available to biological visual systems.

Although it is possible to model how neural activity in different sensory systems could be combined using Bayesian decision theory (Fetsch et al., 2013), such models cannot indicate how information about the physical world could be obtained in a way that avoids the quandary illustrated in Figure 1. Indeed, any model based on recovering or estimating real-world parameters, statistically or otherwise, will fail as a canonical explanation of visual perception (see also Jones and Love, 2011; Bowers and Davis, 2012). Biological vision must therefore depend on some other strategy that does not require accessing the real-world parameters of image sources.

## Information Theoretic Approaches

A different empirical approach to vision is based on information theory. Within a few years of Claude Shannon’s idea of using Boolean algebra to design switching circuits that could make messages transmitted over noisy communication channels more efficient (Shannon, 1948; Shannon and Weaver, 1949), this framework was applied to vision (Attneave, 1954; Barlow, 1961). The premise of these studies was that the properties of visual and other sensory systems would encode, transmit, and decode the empirical characteristics of naturally occurring stimuli with maximum efficiency. Subsequent approaches in these terms have variously interpreted vision to operate on the basis of predictive coding (Srinivasan et al., 1982; Rao and Ballard, 1999; Hosoya et al., 2005); coding that de-correlates the information of noisy inputs (Barlow, 1961; Laughlin, 1981); a filtering scheme for ensuring sparse coding (Olshausen and Field, 1996); and/or greater efficiency achieved by divisive normalization (Schwartz and Simoncelli, 2001; Carandini and Heeger, 2012).

The overarching theme of this approach is that optimizing information transfer by minimizing the metabolic and other costs of wiring, action potential generation and synaptic transfer—while at the same time maximizing the entropy of neural communication—could rationalize the characteristics of receptive fields in visual animals (Graham and Field, 2007). As it has turned out, the idea that some features of visual systems arise from efficiently encoding the statistical structure of natural environments is consistent with a number of computational (Srinivasan et al., 1982; Atick and Redlich, 1993; Olshausen and Field, 1996; Bell and Sejnowski, 1997; van Hateren and van der Schaaf, 1998; Brady and Field, 2000; Schwartz and Simoncelli, 2001; Simoncelli and Olshausen, 2001) and physiological studies (Dan et al., 1996; Baddeley et al., 1997; Vinje and Gallant, 2000).

Although the success of models based on information theory leaves no doubt about the advantages of efficient visual processing, the models do not explain how the inevitable conflation of information in images is dealt with by the visual system (see earlier and Figure 1), or why perceived visual qualities do not correspond with measured physical parameters in the visual environment (see Figure 2). Nor do they indicate how biological visual systems successfully guide behavior.

While these deficiencies do not diminish the importance of efficient neural processing conceived in terms of Shannon entropy, efficiency is not directly germane to perception and behavior, just as efficiency in telecommunication is not germane to the content of the messages that are transmitted. Generating perceptions that succeed in a world whose physical parameters cannot be recovered is a different goal, in much the same way that the functional aim of any organ system differs from the concurrent need to achieve its purposes as efficiently as possible.

## A Wholly Empirical Approach

The aim of the visual system in these approaches is assumed to be the recovery of real world properties, however imperfectly, from information in retinal stimuli. A different supposition is that since retinal images cannot specify the measurable properties of objects (see Figure 1), achieving this goal is impossible. It follows that visual perceptions must therefore arise from a strategy that does not rely on real world properties as such. In a wholly empirical conception of vision, the perceptual values we experience are determined by ordering visual qualities according to the frequency of occurrence of image patterns and how this impacts survival (Purves and Lotto, 2003, 2011; Purves et al., 2011, 2014).

**Figure 3:**
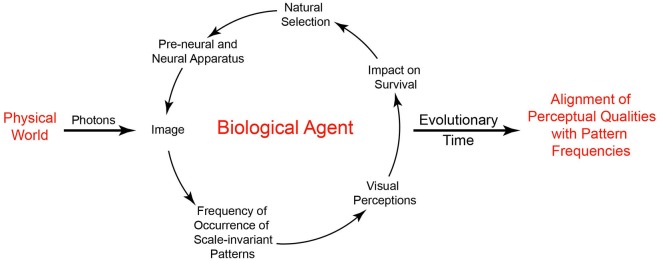
**Visual perception based on the frequency of occurrence of patterns and subsequent behavior.** By depending on the frequency of scale-invariant patterns in images, useful perceptions can arise without information about physically measurable properties of the world. The driving force in this understanding of vision is a biological feedback loop that, over time, orders the basic visual qualities we perceive by associating the frequency of recurring image patterns with perceptual qualities according to survival and reproductive success.

In general terms, understanding this strategy is straightforward. Imagine a population of primitive organisms whose behavior is dictated by rudimentary collections of photoreceptors and associated neural connections. As stipulated by neo-Darwinian theory, the organization of both the receptors and their connections in the population is subject to small random variations in structure and function that are acted on by natural selection. Based on interactions with the environment, variations of pre-neural and neural configurations that promote survival tend to be passed down to future generations. As a result, the ranks of visual qualities an agent perceives over some evolved range (darkest-lightest, largest-smallest, fastest-slowest, etc.) reflect biological utility rather than the physically measureable properties of objects and conditions in the world. In short, the role of perceptual states is not to reveal the physical world, but to promote useful behaviors. In this scheme, the world is simply the arena in which the utility of perceptions and other behavioral responses pertinent to survival and reproduction is tested, with feedback from the environment acting as the driving force that gradually instantiates the needed circuitry (Figure 3).

In implementing this strategy, however, vision cannot rely on entire images, as efficient coding theory has long recognized (see Information Theoretic Approaches). The reason is that the extraordinary detail in most retinal images will rarely, if ever, activate the full array of photoreceptors in exactly the same way again. Processes like evolution and lifetime learning, however, depend on repeated trial and error. Thus rather than relying on images *per se*, biological vision is better served by relying on the recurring scale-invariant patterns within images to rank perceptual qualities (scale invariance refers to a relationship that does not change when variables such as length and width are multiplied by a common factor). In this way the biological feedback loop diagrammed in Figure 3 can progressively organize both ordinal (e.g., lighter-darker, larger-smaller) and non-ordinal (e.g., color, direction) visual qualities over useful ranges according to the relative frequency of pattern occurrences and feedback from behavior. This concept is consistent with classical physiological studies demonstrating the transformation of images by the evolved receptive fields of early level visual neurons (Hubel, 1988; Hubel and Wiesel, 2005), with the goal of reducing the redundancy of image information by efficient coding (Graham and Field, 2007), and with psychophysical studies showing that the frequency of occurrence of image patterns extracted from natural scenes predicts human visual perceptions (Yang and Purves, 2004).

## An Example

**Figure 4:**
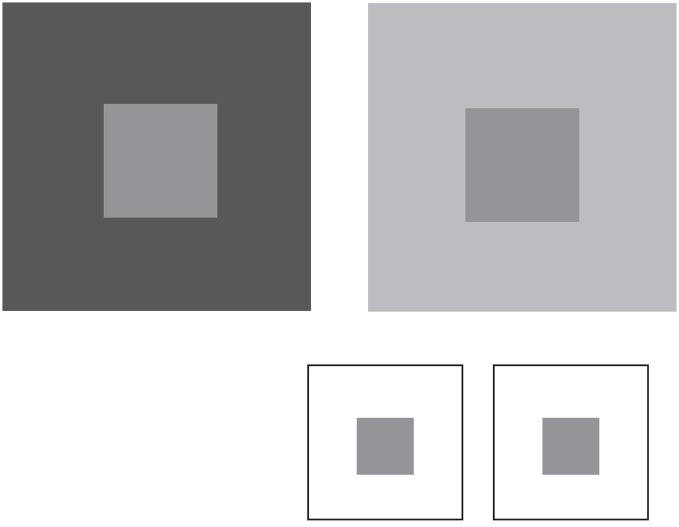
**Lightness percepts elicited by luminance patterns.** The two patterns comprise central squares with identical luminance values surrounded by regions that have a lower (left panel) or higher (right panel) luminance. The central squares appear differently light in these contexts, despite the fact that they are physically the same. The inset shows that when placed on the same background the central squares elicit the same lightness, although this percept differs from the lightness of the squares in either of the two patterns above.

To appreciate how vision can operate in this way, consider the perceptions of lightness-darkness elicited by natural luminance patterns. Figure 4 shows two simple patterns in which the luminance of the central squares is the same, but the luminance of the surrounding areas differs. As has been noted since Michel Chevreul’s studies in the 19th century, the central squares appear differently light, thus failing to agree with physical measurements.

**Figure 5:**
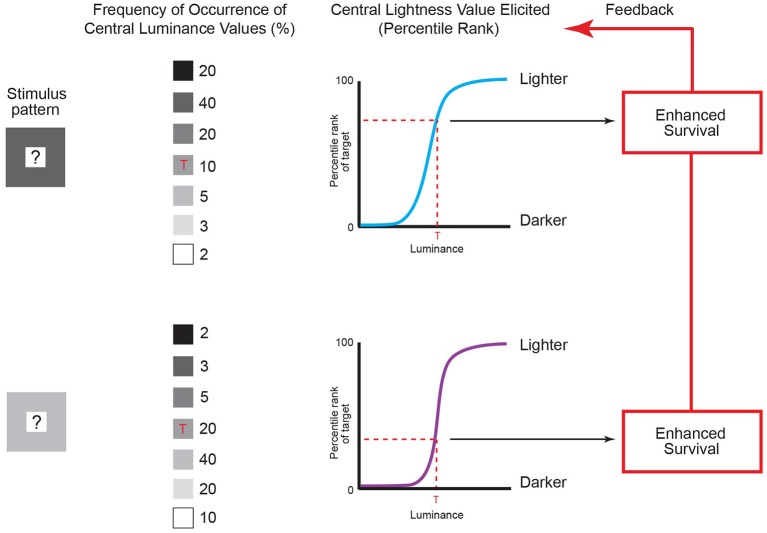
**Lightness predicted by the frequency of recurrent luminance patterns.** The contexts of luminance patterns in column 1 are the same as in Figure 4, with an unspecified central value indicated by the question marks. The frequency of occurrence of central luminance values in these patterns can be determined by repeatedly sampling natural images using the patterns as templates (see column 2). To maximize behavioral success, the lightness elicited by the central luminance value in Figure 4 (indicated by the red ‘Ts’ in column 2) should evolve to accord with its accumulated frequency of occurrence in the two patterns (dashed red lines in the graphs in column 3) rather than with its actual luminance, thus explaining why the same central luminance in Figure 4 is perceived differently. Organisms therefore evolve to match their perceptions to the accumulated frequencies of occurrence of targets given a context through their enhanced survival over evolutionary time (as shown in Figure 3). (Note that using templates to determine the frequency of occurrence of patterns is simply a convenient way of collecting the pertinent data, and does not imply that the visual system uses templates to sample retinal images.) (Original data is in Yang and Purves, 2004).

In wholly empirical terms, the reason for this effect is outlined in Figure 5. In the course of maximizing survival and reproductive success in response to scale-invariant patterns of luminance, evolution and lifetime learning will have ranked perceptions of relative lightness-darkness according to the frequency of occurrence of the luminance of any element in a pattern, given the luminance values of the rest of the elements. Absent this ordering according to the frequency of recurring image patterns, the generation of useful perceptions and behaviors would be stymied by the fact that these or any other patterns cannot specify the measured properties of the objects and conditions that gave rise to them (see Figure 1).

As shown in Figure 5, the empirical incidence of the two patterns arising in retinal images generated by a database of natural images shows that the same central luminance value occurs less often in the context of a lower-luminance surround than in the context of a higher-luminance surround (column 2; Yang and Purves, 2004). The reason is that in any non-random pattern, nearby points will tend to have similar luminance values (see Figure 4; Olshausen and Field, 1996, 2000). Consequently, if the lightness-darkness values of the central squares are ordered according to their relative frequency of occurrence in these patterns (column 3), the same luminance value should elicit a lighter appearance in the context of a less luminant surround when compared to a more luminant surround, as it does (see Figure 4).

In summary, the frequencies of occurrence of luminance values in image patterns responded to over time predict the qualities we see in this example because the range of this basic perceptual quality (lightness-darkness) has been ordered over a useful range (lightest to darkest) according to the relative success of stimulus-response associations. Similar ordering of data arising from the frequency of pattern occurrence in both natural and simulated environments has been used to rationalize more complex stimuli that elicit perceptions of lightness (Yang and Purves, 2004), color (Long et al., 2006), interval and angle magnitude (Howe and Purves, 2005), the speed of motion (Wojtach et al., 2008, 2009), and the direction of motion (Sung et al., 2009).

## Consequences of Input-Output Associations on a Wholly Empirical Basis

Behaviorally successful associations generated in this way automatically tie the frequency of occurrence of stimulus patterns to the frequency of occurrence of responses, explaining why relying on the frequency of occurrence of stimulus patterns predicts perception: every time a given image pattern occurs as input, the associated output arises from trial and error feedback, which in biology tracks reproductive success. The result is perceptions that become more and more useful over time. Although in any trial and error process input-output equivalence is never reached, after sufficient evolution the cumulative distribution function of the stimulus input will come to align with the cumulative distribution function of the perceptual output closely enough to predict many of the results of human psychophysics (Purves et al., 2014).

When conceived in this way it makes sense that visual perceptions are not correlated with light intensity or any other physical property, as psychophysics amply demonstrates. Although relying on the frequency of occurrence of patterns uncouples perceived values from their measured physical parameters (e.g., surface reflectance), it endows visual agents with the ability to perceive and act in their environments in ways that led to biological success in the past, and are therefore likely to succeed in the present. While this strategy makes it seem that we see the world as it really is, vision on a wholly empirical basis is not veridical and has a different goal: to generate useful perceptions without measuring or recovering real-world properties.

## Exploring Neuronal Connectivity in Wholly Empirical Terms

Bayesian approaches to perception use inferences about real-world properties as a tool for understanding whatever processing is accomplished by the visual brain. But as has already been emphasized, biological sensing systems cannot recover these properties.

The wholly empirical alternative we describe is generally consistent with other studies that do not assume the recovery of real-world properties (e.g., Janke et al., 1999; Onat et al., 2011). Ultimately, any approach to vision based on empirically successful input-output associations must explain how this strategy is related to the documented physiology and anatomy of the primate and other visual systems. In principle, the most direct way to unravel the circuit mechanics underlying a wholly empirical (or any other) strategy would be to mimic the trial and error process of association on which evolution relies. Until relatively recently, this approach would have been fanciful. But the advent of genetic and other computer algorithms has made simulating the evolution of artificial neural networks in model environments relatively easy. This technology offers a way of linking any empirical understanding of vision to the wealth of information already in hand from physiological and anatomical studies.

A number of studies have shown the feasibility of evolving neural networks on the basis of experience (Geisler and Diehl, 2002; Boots et al., 2007; Corney and Lotto, 2007; Geisler et al., 2009; Burge and Geisler, 2011). More recent work has asked whether the connectivity and operating principles of networks evolved on a wholly empirical basis is similar to that found in biological circuitry. For example, simple networks have been evolved to rank responses according to the frequency of occurrence of patterns extracted from natural and simulated images (Ng et al., 2013; Morgenstern et al., 2014). The most obvious feature that emerges is the center-surround receptive field. In addition to efficiency, this organization enables the interaction of targets and contexts, heightens sensitivity to frequently occurring stimuli, and automatically adapts to overall luminance and local contrast. These features are all characteristic of neurons in the early stages of visual systems like ours (Sakmann and Creutzfeldt, 1969; Geisler and Albrecht, 1992; Bonin et al., 2005; Hubel and Wiesel, 2005).

## Vision as Reflexive

Any fully empirical account of vision implies that perceptions and their neural underpinnings are reflexive. The term “reflex” alludes to behaviors such as the “knee-jerk” (myotatic) response that depend on the transfer of information from sensory input to motor output via circuitry established by behavioral success over evolutionary time. The advantages of reflex responses are clear: circuitry that links input to output as directly as possible allows the nervous system to respond with maximum speed and accuracy. It does not follow, however, that reflex responses must be “simple”, that they are limited to motor acts, or that they entail only “lower order” neural circuitry. Sherrington (1947), who pioneered the study of reflex circuits, was well aware that the concept of a “simple” reflex is, in his words, a “convenient…fiction”, since “all parts of the nervous system are connected together and no part of it is ever capable of reaction without affecting and being affected by other parts …”. There is no evidence that any response to sensory input differs from a spinal reflex, other than by the number of synaptic connections in the input-output circuitry. Understanding vision as reflexive (i.e., hard-wired at any given moment but subject to modification by subsequent experience) also affords the ability to account for visual perceptions generated within a few tens of milliseconds in response to complex stimuli such as wind-blown leaves, running water, animal movements and numerous other circumstances. Computer vision models that depend on reverse-engineering scenes from images by inferring the large number of real world sources that could have generated these complex image streams would likely require more computational power than is necessary for the tasks that visual and other biological sensing systems routinely carry out. Although it is difficult to imagine how visual systems could generate perceptions of complex scenes almost immediately by a series of hierarchical computations, this problem is resolved if visual “processing” is re-imagined as the result of “computations” that have, in effect, already been accomplished by laying down connectivity instantiated by feedback from empirical success over evolutionary and individual time (see Figure 3). This strategic difference is presumably the main reason why machine vision based on logical algorithms cannot match the performance of biological vision on many tasks.

## Limitations of a Wholly Empirical Approach

As with any theory, there are limitations to the strategy of visual perception advocated here, both methodological and conceptual. With respect to methodology, when investigating the perception of lightness (see Figures 4, 5), the luminance values comprising the database were collected from a limited range of environments assumed to be representative of the types of scenes where the human visual system evolved. In addition, the fact that humans and other animals attend to specific aspects of the environment, thus biasing the frequency distribution of sensory input, was not taken into account. While these and other deficiencies are important, given that this strategy successfully predicts the standard simultaneous lightness contrast effect shown in Figure 4 and a variety of more complex lightness effects (Yang and Purves, 2004)—in addition to other puzzling perceptions of color, form and motion (see above)—the empirical framework seems well supported by evidence that has not been supplied by other approaches. This last point stands as a challenge to any theory of perception, including broader unifying concepts such as the idea that the common goal of brain function is to satisfy a “free-energy principle” (Friston, 2010).

## The Wholly Empirical Theory and Cognition

It is worth noting that higher order phenomena such as visual attention and visual memory could also arise by associating the relative frequency of recurring scale-invariant image patterns with useful responses. As in the case of the basic visual qualities considered here, the relevant circuitry would also be reflexive, without the need to invoke additional “cognitive” mechanisms: every time a given image pattern occurred the response dictated by association would be further enhanced according to its utility. As a result the foci of visual attention and the visual memories elicited would, like perceptions, gradually become more and more useful over time.

## Conclusion

The idea that vision operates empirically has taken several forms and enjoyed different degrees of enthusiasm since Helmholtz introduced the concept of unconscious inference in the 19th century. Vision on a wholly empirical basis is now seen by some investigators as the most plausible way to understand how stimuli that cannot specify their physical sources can nonetheless give rise to useful perceptions and routinely successful visually guided behaviors. Understanding perception in these terms implies a strategy of nervous system operation that differs fundamentally from the concept of detecting stimulus features and recovering real-world properties by algorithmic computations that in one way or another depend on accessing physical parameters to guide actions. By relying on evolved reflex associations that have ordered visual qualities according to the impact of the relative frequency of occurrence of stimulus patterns on reproductive success, vision can circumvent the inherent uncertainty of retinal images, and explain the qualities we actually see.

## Conflict of Interest Statement

The authors declare that the research was conducted in the absence of any commercial or financial relationships that could be construed as a potential conflict of interest.
